# Expired breath condensate hydrogen peroxide concentration and pH for screening cough variant asthma among chronic cough

**DOI:** 10.4103/1817-1737.30357

**Published:** 2007

**Authors:** Amina Hamed Ahmad Al Obaidi

**Affiliations:** *Department of Biochemistry, College of Medicine, Tikrit University, Tikrit, Iraq*

**Keywords:** Asthma, chronic cough, cough variant asthma, hydrogen peroxide, pH

## Abstract

The reported studies suggest a role for eosinophils in the pathogenesis of cough variant asthma. In the present study, the expired breath condensate level of hydrogen peroxide and pH were determined in patients with cough variant asthma and compared to subjects with classical asthma, with chronic cough nonasthmatic and healthy control. Twenty-seven patients with cough variant asthma, 43 patients with classical asthma, 32 patients with chronic cough and 27 healthy subjects were studied in a cross-sectional study. Hydrogen peroxide concentration was significantly higher in cough variant asthma as compared to chronic cough nonasthmatic patients and healthy control subjects, while pH level was significantly lower in cough variant asthma as compared to chronic cough nonasthmatic patients and healthy control subjects. However, there was no significant difference in expired breath condensate hydrogen peroxide and pH between cough variant asthma and classical asthma. In conclusion, hydrogen peroxide concentration and pH of expired breath condensate may be used as noninvasive markers to differentiate cough variant asthma from chronic cough.

Chronic nonproductive cough resistant to antibiotics and the usual antitussive agents is a common problem in the pulmonary clinics.[1] Of those patients whose chronic cough goes undiagnosed, 30 -50% of those patients have unrecognized or cough variant asthmatics.[2] However, cough may be the sole manifestation of asthma.[3,4] Corrao *et al*[4] in 1979 reported that six subjects complaining of chronic persistent cough without wheezing or dyspnea and their cough soon disappeared after starting either bronchodilators but recurred when they were stopped. The authors regarded these subjects as having a variant form of asthma, which was named, cough variant asthma (CVA). Several reports on CVA followed then and the condition now recognized as a common cause of chronic cough.[5,6]

Recent research has clarified that asthma is a chronic inflammatory airway disease, in which eosinophils play a central role,[7] consequently mediators released that lead to bronchoconstriction.[7,8] Previous studies indicated increase in eosinophils in peripheral blood, sputum, bronchoaleveolar lavage fluid and bronchial mucosa.[8–11] The intensity of eosinophilic inflammation correlates with clinical severity of the disease, bronchial hyperresponsiveness and lung function.[8–11] Only four studies, to our knowledge, have examined the relationship between CVA and inflammation.[3,10,12,13] These reports suggest a role of eosinophils in the pathogenesis of CVA.

Activation of inflammatory cells and particularly eosinophils, is the prominent feature of airway inflammation in patients with asthma.[14] Eosinophils release several mediators, including hydrogen peroxide that may amplify the inflammatory process in the airways.[15] Eosinophils are activated both locally in the lungs and in the blood.[16] As a consequence for hydrogen peroxide release, tracheal smooth muscle stimulated and contract, followed by stimulation of cough receptors to induce cough.[17]

There is a relatively close association between inflammation and low pH which is shown by the further fall in pH during exacerbation.[18] The drop in airway pH before and during asthma exacerbation would cause extensive eosinophils necrosis with an acute release of inflammatory and bronchoconstricting products,[19] followed by induction of cough. Thus exhaled breath condensate acidification could be representative of an airway pH homeostatic alteration that underlies some or much of the pathology of lung diseases including inflammation in asthma.[20] The hypothesis that was suggested, there was a positive association between exhaled breath condensate hydrogen peroxide concentration and pH in cough variant asthma and present in chronic cough patient. Therefore, utility of these two noninvasive markers of airway inflammation was evaluated in cough variant asthma and have compared to their levels in patients with classical asthma chronic cough and healthy control.

## Materials and Methods

### Patients

Twenty seven patients with CVA, 43 patients with classical asthma, 32 patients with chronic cough and 27 healthy subjects were studied in a cross-sectional study. The study was performed during the period from April 2005 to August 2006. The patients with CVA referred to Allergy and Asthmology Center in Tikrit Teaching Hospital for chronic cough persisting for longer than two months duration but without wheezing or dyspnea. They had no past history of asthma or other respiratory diseases. Wheeze or rhonchi were not audible on chest auscultation even at forced expiration. Bronchodilators (inhaled B agonist and/or oral theophyllines) were effective against their cough. No other apparent causes of cough were present, they did not have any sign or symptoms of postnasal drip or gastro – oesophegeal reflux, had not taken angiotensin converting enzyme inhibitors and had normal chest radiograph results.

The patients with classic asthma had a history of episodic dyspnea, wheezing and cough. They had at least 15% reversibility of forced expiratory volume in one second (FEV1) after inhalation of 200 μg of salbutamol. The control subjects had no past history of asthma or other respiratory diseases and had no current respiratory symptoms. Each patient with CVA or classic asthma had been treated with inhaled B agonist (used as needed) and/or theophylline taken twice daily. Treatment with B agonist was withheld for eight hours prior to the test of FEV1. Treatment with theophylline was withheld for 48 hours prior to all examinations. None of the studied subjects had ever taken systemic corticosteroids, cromoglycate or other antiallergic agents, had smoked within the previous two years or had respiratory infections for the last eight weeks. Thirty two patients with chronic cough and not asthmatic were included as chronic cough control. Asthma diagnosis was according to National Heart, Lung and Blood Institute guidelines for the diagnosis and management of asthma.[21]

The study was approved by the ethics committee of our college and written consent was obtained from all participating subjects.

### Lung function test

Lung function was measured by dry spirometry.

### Hydrogen peroxide measurement

Expired breath condensate was collected by using a glass condensing device that was placed in a large chamber with ice. After rinsing their mouth, subjects breathe tiedly with normal frequency through a mouthpiece for 20 minutes while wearing a nose clip. H_2_O_2_ assay was carried out by using colorimetric assay as described previously.[22] Briefly, 100 μl of condensate was mixed with 100 ml of tetramethylbenzidine in 0.42 mol/l citrate buffer, pH 3.8 and 10 μl of horseradish peroxidase (52.5 U/ml). The samples were incubated at room temperature for 20 minutes and reaction was stopped by addition of 10 μl 18N sulfuric acid. The reaction product was measured spectrophotometrically at 450 nm. A standard curve of H_2_O_2_ was performed for each assay.

### pH Measurement

Expired breath condensate pH was measured as previously described[19] right after the collection of the condensate by using a pH meter (HI 8424).

### Statistical analysis

Data concerning the comparisons among the various parameters in the study groups are given as mean (SD) with 95% confidence intervals for the differences. Student's unpaired two-tailed test was used for significant testing.

## Results

Expired breath condensate hydrogen peroxide in cough variant asthma was significantly higher (0.83 ± 0.34 μmol, 95% CI 0.69-0.96 μmol, *P*<0.0001), than that for nonasthmatic chronic cough (0.24 ± 0.22 μmol, 95% CI 0.16-0.32 μmol) and healthy control (0.29 ± 0.07 μmol, 95% CI 0.27-0.31 μmol, *P*<0.0001). However, there was no significant difference in exhaled breath condensate H_2_O_2_ concentration between cough variant and classical asthma [Table 1].

**Table 1:** Expired breath condensate hydrogen peroxide concentration and pH in cough variant asthma

Index	Healthy control[23]	Classical asthma[24]	Chronic cough[25]	Cough variant asthma[26]
Hydrogen peroxide	0.29	0.89	0.24	0.83
Mean	0.07	0.36	0.22	0.34
SD	0.27-0.31	0.78-1.00	0.16-0.31	0.69-0.96
95% Cl				
pH				
Mean	7.82	5.97	7.32	6.26
SD	0.65	1.03	0.60	0.43
95% Cl	7.61-7.99	5.65-6.29	7.10-7.54	6.09-6.43

The exhaled breath condensate pH was significantly lower in cough variant asthma (6.26 ± 0.43, 95% CI 6.09-6.43, *P*<0.0001) than that for nonasthmatic chronic cough (7.32 ± 0.6, 95% CI 7.1-7.54) and healthy control (7.82 ± 0.65, 95% CI 7.61-7.99, *P*<0.0001). However, pH was lower in classical asthma as compared to that in cough variant asthma, but the difference did not reach significant value.

H_2_O_2_ exhaled breath concentration and pH not significantly different between healthy control subjects and nonasthmatic patients with chronic cough.

## Discussion

The inflammatory cells recruited to the asthmatic airways have an exceptional capability for producing reactive oxygen species including hydrogen peroxide.[26] The oxidative injury caused by eosinophils can be substantial because the cells possess several times greater capacity to generate hydrogen peroxide than neutrophils.[27] The reported studies indicated that hydrogen peroxide concentrations and eosinophils numbers were increased in exhaled breath condensate of asthmatic patients.[15,25,28–30]

Cough variant asthma was first described by Glauser in 1972.[31] The only presenting symptoms are isolated chronic cough responsive to bronchodilator treatment.[32] Recognition of cough variant asthma is clinically important because bronchodilator treatment is only effective in cough variant asthma and can be prevented from progression to classical asthma. Bronchodilators usually exert no antitussive effect in other cases of isolated chronic cough.[33] Thus for the purpose of effective treatment of chronic cough, there was a need for differentiating cough variant asthma from other cough entity.

In the present study, patients with cough variant asthma show high level of expired breath condensate H_2_O_2_ than that in patients with chronic cough nonasthmatic and healthy control. Furthermore, expired breath condensate pH was lower in cough variant asthma than that in patients with chronic cough nonasthmatic subjects and healthy control. However, the expired breath condensate H_2_O_2_ and pH in cough variant asthma were not significantly different from values for patients with classical asthma. In addition, expired breath condensate H_2_O_2_ and pH were not with significant difference between healthy control and patients with nonasthmatic chronic cough. The increased expired breath condensate H_2_O_2_ and decreased pH indicate the involvement of eosinophilic inflammation in cough variant asthma, as well as confirm the results of previous studies on the relationship between eosinophils and classic asthma.[3] This was suggested depending on that patients with CVA have a high number of eosinophils in bronchoalveolar lavage fluid and bronchial tissue and increased serum eosinophil cationic protein as compared to healthy control.[3] In addition, activation of inflammatory cells and particularly eosinophils, is a key feature of asthmatic inflammation[14,34] and may be reflected by increased hydrogen peroxide concentration in exhaled breath condensate.

The correlation of inflammation to cough in cough variant asthma is not clear. O'Conell *et al*[6] have hypothesized that patients with cough variant asthma might have inflammation solely in the large airways, where cough receptors are abundant. Woolcock[35] has also stated that cough receptors are assumedly stimulated by the inflammatory process and the cough in cough variant asthma will be mediated by in the central ways. Niimi *et al*[3] performed bronchial biopsy at segmental bronchi, relatively central airways and broncho aleveolar lavage, which might reflect the inflammation of the whole airways from sub segmental bronchi to alveoli. Both procedures, however, revealed no differences between classic asthma and cough variant asthma and this evidence that strengthen our finding in this study. The site of inflammation may not be causally related to the difference in presenting manifestations from these results.[3] Examinations at more central airways are required in a future study to elucidate this issue.[3] Tussive mediators which are secreted from eosinophils[7] may be possible mechanisms for the induction of cough in cough variant asthma.[7,36]

Another studies reported on several patients with chronic cough with normal pulmonary function tests and normal bronchial responsiveness.[37] These patients showed an increase in eosinophil as well as metachromatic cells in sputum. After treatment with inhaled corticosteroids, cough decreased markedly and bronchial responsiveness improved in some patients. The authors labeled these patients as “eosinophilic bronchitis without asthma”. The presence of bronchial responsiveness in cough variant asthma but not in eosinophilic bronchitis in the studies of Gibson *et al*[37,38] is difficult to explain, since eosinophilic inflammation, an essential cause of bronchial responsiveness in asthma,[7–9] is present in both conditions. The differences in the intensity of inflammation, degree of activation of eosinophil, the initial level of bronchial responsiveness,[3] the location of inflammation[37,38] may influence the variation between two conditions. These suggestions may explain the differences in expired breath condensate H_2_O_2_ and pH between cough variant asthma and chronic cough patients as this study indicated.

The mechanisms of cough in asthma have been discussed in view of airflow obstruction.[3] It was proposed that cough receptors are stimulated by local bronchoconstriction.[17] As bronchodilators are effective against cough in cough variant asthma, this may be a possible pathogenetic mechanism.[39] Cough may be the predominant symptom during exacerbation in some asthmatic patients.[40] Pulmonary function test revealed narrowing of the central airways, whereas another group of asthmatics, who predominantly complained from dyspnea, had narrowing of the peripheral airways, thus the predominance of cough was due to central airway obstruction.[40] Other studies on cough variant asthma, however, showed almost normal pulmonary function[4,5,41] or peripheral rather than central airway obstruction[42] at baseline. Examinations after methacholine challenge[4] or exercise[41] revealed obstruction of both central and peripheral airways, as seen identically in classic asthma[4] or obstruction predominantly the peripheral airways.[41] In the study of Niimi *et al*,[3] the parameters of baseline pulmonary function, including indices of both central and peripheral responsiveness, did not significantly differ between classic and cough variant asthmatic patients.

The finding of the present study was in consistent with that of Niimi,[3] which indicate no significant difference in expired breath condensate H_2_O_2_ and pH as markers of inflammation and oxidative stress between cough variant and classic asthmatic patients. It is thus not certain whether the site of airflow obstruction or degree of bronchial responsiveness[43] is causally related to differences in presenting manifestations. One possible mechanism for cough without wheeze in cough variant asthma is proposed by Koh *et al*.[43] They demonstrated a higher wheezing threshold to inhaled methacholine in cough variant asthma than in classic asthma. Heightened cough receptor sensitivity may also be a mechanism [36] but some negate this possibility[39] and the issue is still controversial.[3]

The prevalence of asthma is increasing worldwide.[1] However, the disease is still under-diagnosed.[44] Recognition of cough variant asthma in the differential diagnosis of patients with chronic cough is quite important from clinical standpoint[4–6,28] although it's pathogenetic have not yet been fully elucidated.[3] Appropriate diagnostic procedures, including detection of inflammatory markers in expired breath condensate should be considered. The presence of elevated H_2_O_2_ and reduced pH of expired breath condensate, which may suggest eosinophilic airway inflammation and possible diagnosis of cough variant asthma, may be of some help in the initial assessment of patients with chronic cough. Irreversible pathological changes of airway in asthmatic patients due to under treatment of the disease, is assumed to be due to persistent inflammation.[24] Such irreversible changes may occur also in cough variant asthma, since inflammation is present in the airways of patients with cough variant asthma, as demonstrated in this study. Early introduction of inhaled corticosteroids may lead to better prognosis in cough variant asthma, as reported for classic asthma.[45] However, this suggestion needs to be investigated in future studies.

Nearly 30% of patients with cough variant asthma may develop classic asthma.[4,23] It is noteworthy that the rate of onset of typical asthma was significantly lower in patients treated with beclomethasone dipropionate inhaler, which suggests the usefulness of long term inhaled steroids as an intervention against cough variant asthma. Thus early diagnosis and intervention are important in controlling asthma.[21] As cough variant asthma is thought to be precursor of typical asthma, early intervention for cough variant asthma may be as important as in mild asthma.[32] A diagnosis of cough variant asthma has been made based on the efficacy of steroids by some investigators,[46–49] but steroids are effective not only in cough variant asthma but also in atopic cough.[32] It is therefore likely that the use of steroids efficacy as a criterion to diagnose cough variant asthma may accidentally misclassify patients who actually have atopic cough.[32] This may account for the increased cough sensitivity in patients with cough variant asthma studied by McGarvey and colleagues.[50]

## Conclusion

Determination of hydrogen peroxide concentration and pH of exhaled breath condensate indicate that they were significantly different between cough variant asthma and chronic cough in nonasthmatic patients. However, there were no significant differences between cough variant asthma and classical asthma in relation to values of hydrogen peroxide and pH in expired breath condensate. Thus it was recommended that it is important to use pH and H_2_O_2_ expired breath condensate concentration as a criterion to help in diagnosis of cough variant asthma.
